# Inhibition of KDM4A restricts SQLE transcription and induces oxidative stress imbalance to suppress bladder cancer

**DOI:** 10.1016/j.redox.2024.103407

**Published:** 2024-10-22

**Authors:** Jiapeng Zhang, Hang Xu, Yirui He, Xiaonan Zheng, Tianhai Lin, Lu Yang, Ping Tan, Qiang Wei

**Affiliations:** a Department of Urology, Institute of Urology, Sichuan Clinical Research Center for Urological and Kidney Diseases, West China Hospital, Sichuan University, Chengdu, China; b Department of Hematology, West China Hospital, Sichuan University, Chengdu, China

**Keywords:** Kdm4a, ML324, Bladder cancer, Sqle, ROS, Oxidative stress

## Abstract

In clinical practice, the limited efficacy of standard comprehensive therapy for advanced bladder cancer and the lack of targeted treatment options are well recognized. Targeting abnormal epigenetic modifications in tumors has shown considerable potential in cancer therapy. Through drug screening in tumor organoids, we identified that ML324, a histone lysine demethylase 4A (KDM4A) inhibitor, exhibits potent antitumor effects in both in vitro and in vivo cancer models. Mechanistically, Kdm4a demethylates H3K9me3, leading to chromatin opening and increased accessibility of Gabpa to the squalene epoxidase (Sqle) gene promoter, resulting in transcriptional activation. Inhibition of Kdm4a downregulates Sqle transcription, blocking cholesterol synthesis and causing squalene (SQA) accumulation. This process induces reactive oxygen species (ROS) clearance and suppresses JNK/c-Jun phosphorylation, ultimately inducing apoptosis. Furthermore, ML324 treatment significantly inhibited tumor growth in bladder cancer patient-derived xenograft (PDX) models. Our findings reveal the presence of a Kdm4a-Sqle-ROS-JNK/c-Jun signaling axis that regulates oxidative stress balance, offering a novel strategy for targeted therapy in bladder cancer.

## Highlights

• Inhibition of Kdm4a downregulates Sqle by disrupting Gabpa-mediated transcriptional activation.
● Sqle deficiency leads to squalene accumulation, reducing intracellular ROS levels.
● ROS reduction decreases JNK/c-Jun phosphorylation, promoting apoptosis.
● The KDM4A-SQLE-JNK/c-Jun axis is consistently observed in bladder cancer PDX models.

## Graphical abstract

**Figure:**
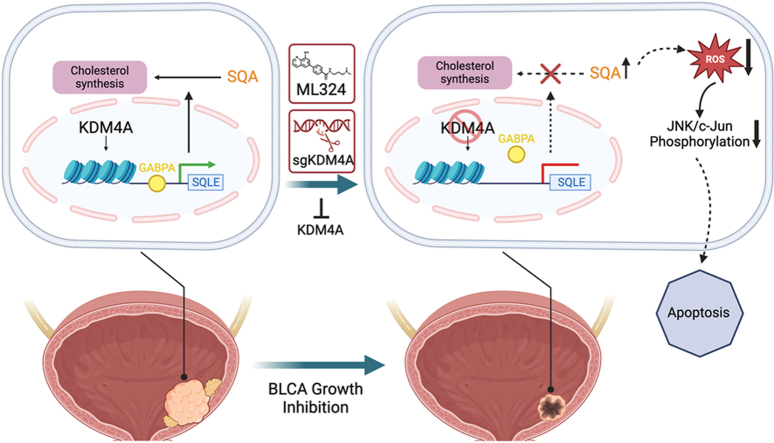

## Abbreviations

KDM4A: Histone lysine demethylase 4A
SQLE: Squalene epoxidase
SQA: Squalene
ROS: Reactive oxygen species
PDX: Patient-derived xenograft
BLCA: Bladder cancer
MIBC: Muscle-invasive bladder cancer
FPP: Farnesyl pyrophosphate
2,3-OxS: 2,3-oxidosqualene
LAN: Lanosterol
NSG: NOD scid gamma
IHC: Immunohistochemistry
TF: Transcription factors
TSS: Transcription start site

## 1 Introduction

Bladder cancer (BLCA) is a common malignancy of the urinary system, with high-grade muscle-invasive bladder cancer (MIBC) posing a significant therapeutic challenge due to its aggressive nature and high recurrence rates [1,2]. Although surgical intervention combined with chemotherapy or radiotherapy has provided certain benefit, the recurrence and resistance rates remain high, and long-term survival outcomes have not significantly improved [3]. Moreover, targeted therapies for bladder cancer have shown limited efficacy. Current strategies, including anti-angiogenic agents like bevacizumab, EGFR inhibitors, and FGFR inhibitors, have demonstrated modest efficacy in certain patient subsets, but overall response rates are low, and resistance frequently emerges [[4], [5], [6]]. This underscores the urgent need for new therapeutic targets for bladder cancer treatment.

Epigenetic modifications offer a promising avenue as therapeutic targets due to their reversible and regulable nature. The frequent occurrence of abnormal epigenetic modifications in bladder cancer presents opportunities for drug discovery and provides a potential platform for the development of novel targeted therapies [[7], [8], [9]]. Targeting epigenetic regulators, such as histone-modifying enzymes and DNA methylation pathways, could potentially disrupt tumor progression, making epigenetic modulation an important future direction for bladder cancer treatment [[10], [11], [12]].

Metabolic reprogramming is a hallmark of tumor cells, with cholesterol metabolism reprogramming being increasingly recognized as a key aspect of cancer biology. Imbalances in cholesterol homeostasis have been reported in various cancers [13] In brief, increased cholesterol synthesis supports rapid proliferation, maintains membrane structure, and facilitates signaling pathways [14,15], thereby promoting tumor progression. In bladder cancer, enhanced cholesterol synthesis has been linked to tumor metastasis [16]. Metabolic alterations in tumors are largely driven by epigenetic modifications, including histone methylation, which regulates metabolic reprogramming [17,18]. Recent evidence suggests that certain translocated enzymes or metabolites may serve as substrates or cofactors, influencing chromatin modification states [19]. However, the precise regulatory relationship between epigenetic modifications and metabolic reprogramming in bladder cancer, particularly regarding enhanced cholesterol synthesis, and its impact on tumor growth when disrupted, remains unclear.

In our screening for antitumor agents in bladder cancer, we identified the KDM4A inhibitor ML324 as a potent candidate. We found that in the absence of Kdm4a, impaired cholesterol synthesis is primarily due to chromatin condensation leading to transcriptional downregulation of Sqle. This, in turn, resulted in the accumulation of SQA, which scavenged ROS and reduced the activation of the pro-proliferative JNK/c-Jun pathway, and ultimately triggers apoptosis in bladder cancer cells.

## 2 Methods and materials

### 2.1 Bladder cancer organoid culture

MIBC patients-derived organoids were generated under sterile conditions, tumor tissue specimens were thoroughly washed and cut into 1 mm fragments. Subsequently, enzymatic digestion was carried out using a digestion solution (containing 5 mg of collagenase I and 2.5 mg of collagenase IV dissolved in DMEM/F12 medium supplemented with 1 % penicillin-streptomycin). The digestion process was conducted at 37 °C for 1 h with intermittent vortexing every 15 min. Following digestion, the resulting suspension was filtered through a 70 μm filter, and the filtrate was centrifuged to obtain a cell pellet. The cell pellet was resuspended in MatrixGel (Corning) and plated in a 48-well plate. After allowing the MatrixGel to solidify at 37 °C for 30 min, human bladder cancer organoid culture medium was added.

### 2.2 Construction of PDX models

This research was approved by the Ethical Research Committee of West China Hospital under approval numbers 2019-933 and 2020-330. Fresh tumor samples were obtained from patients with MIBC who underwent radical cystectomy at the Department of Urology, West China Hospital, Sichuan University. The specimens were trimmed to 2 mm. Aseptic inoculation needles were filled with tissue fragments and then subcutaneously injected into the bilateral axillary regions of NOD scid gamma (NSG) mice under isoflurane anesthesia to complete the transplantation process.

Within 8-week, NSG mice harboring subcutaneous tumor proliferation were euthanized. Tumor specimens were subsequently segmented, and re-implanted into new NSG mice, completing passage of PDX in vivo. By the 3rd passage, PDX models were ready for in vivo efficacy evaluation of drug intervention.

### 2.3 Organoid based drug screening platform

Mouse bladder cancer organoids were seeded in a 96-well plate at a density of 5000 cells/well. After 24 h, 276 small molecule inhibitors were added to each well at a final concentration of 10 μM. Following a 72-h drug intervention period, bright-field images of the organoids under different treatments were captured an Olympus microscope. The OD values of blank wells, control wells, and drug-treated wells were recorded after incubating with CCK8 reagent for 1 h to characterize changes in cell viability induced by different drugs.

### 2.4 Gene editing

Gene knockout was accomplished by CRISPR/Cas9 system. sgRNAs were cloned into the pLentiV2 vector, which was then packaged using the lentivirus system. The lentiviral supernatant was collected and mixed with TrypLE-digested organoid suspension. After centrifugation for 1h and subsequent 2h incubation at 37 °C, with the assistance of polybrene, the infection was completed. Later, the cells were mixed in MatrixGel and seeded onto 48-well plates. Genomic mutations at the respective sgRNA target sites were determined using the T7E1 assay on the 2nd generation post-infection, to assess the completion of gene knockout.

### 2.5 Orthotopic bladder cancer model

Mouse bladder cancer organoids were digested and counted. Approximately 5000 cells per mouse were mixed with 15 μl of DPBS and 15 μl of MatrixGel and kept on ice. Mice were fasted for 12 h prior to the procedure to empty the bladder. Under isoflurane anesthesia, a lower abdominal incision was made layer by layer to fully expose the bladder. The organoid suspension was carefully injected into the muscular layer of the bladder using an insulin syringe.

Utilizing luciferase carried in the TPM, fluorescence imaging was performed after intraperitoneal injection of the luciferase substrate. Bioluminescent signals within the bladder area were detected by IVIS® Lumina III (PerkinElmer) system, as a proxy for proliferated tumor cells. Approaches above were employed to assess in vivo inhibitory effect of drug interventions on primary orthotopic bladder cancer.

### 2.6 Filipin III staining

Following the disposal of organoid cultures, the organoids were fixed overnight at room temperature in 4 % paraformaldehyde. Subsequently, the fixed organoids were stained with Filipin III dye (50 μg/ml) for 45 min. Filipin III-positive staining intensity and proportion within tumor organoids in different groups were captured by LSM900 (Zeiss), to assess alterations in intracellular free cholesterol levels.

### 2.7 Flow cytometry for ROS and apoptosis detection

For detecting ROS levels, cells from different groups, either at the second-generation post-stable infection or after 1 h of NAC treatment, were harvested and digested. ROS levels were measured using the DCFH-DA probe following the ROS detection kit instructions. For apoptosis detection, cells at the second-generation post-stable transfection or after 16 h of treatment with different concentrations of SQA were harvested, gently resuspended in binding buffer, and stained with Annexin V-FITC and DAPI for flow cytometric analysis.

### 2.8 Chromatin Immunoprecipitation (ChIP) assay

The ChIP assay was performed according to the ChIP kit protocol. Briefly, Kdm4a knockout or control TPM cells, at the second-generation post-stable transfection, were cross-linked and quenched. Cells were incubated overnight with 4 μg of specific antibodies (anti-H3K9me3, anti-H3K9me2 or anti-Gabpa, listed in Table S1). Purified DNA was then used for subsequent ChIP-qPCR and ChIP-PCR to amplify the Sqle promoter region (primer sequences listed in Table S2). Results were normalized to input DNA and IgG control.

### 2.9 Dual-luciferase reporter assay

Potential Gabpa binding sites within the Sqle promoter region were predicted using the JASPAR database (https://jaspar.elixir.no/). Mutations were introduced into sequences fully matching the Gabpa motif, and wild-type and mutant sequences were cloned into pGL3 vectors carrying the Firefly luciferase gene. The pcDNA3.1-basic plasmid was used as a control, while pcDNA3.1-Gabpa was used for Gabpa overexpression. TPM cells were co-transfected with pcDNA3.1 (either basic or Gabpa overexpression) and pGL3 constructs (expressing wild-type or mutant Sqle promoter region sequences). According to the dual-luciferase assay protocol, Renilla luciferase was used as an internal control, and luciferase activity was calculated based on different co-transfection conditions.

### 2.10 Liquid chromatography-mass spectrometry (LC-MS) for metabolite detection

The levels of upstream cholesterol synthesis metabolites in TPM cells under different treatments were measured using LC-MS. Metabolites were extracted by adding methanol to lysed cells, and the supernatant was collected after centrifugation at 15,000g for 10 min at 4 °C. LC-MS was then used to quantify SQA, farnesyl pyrophosphate (FPP), 2,3-oxidosqualene (2,3-OxS), and lanosterol (LAN) levels, with standard curves used for quantification. Metabolite levels in the control group were normalized to 1, and changes in metabolite levels due to Kdm4a knockout were expressed as fold changes.

### 2.11 Statistics

SPSS Statistics v22 was used for data statistics and statistics were expressed as mean ± SD. Statistical analyses of differences in data between groups were performed using Student's t-test or one-way ANOVA, depending on the purpose. Statistical differences were expressed as p value, ∗p < 0.05, ∗∗p < 0.01, ∗∗∗p < 0.001. Graphpad Prism8 software was used to visualize the presentation of statistical data.

## 3 Result

### 3.1 Epigenetic drug library screening based on mouse bladder cancer organoids

**Fig. 1:**
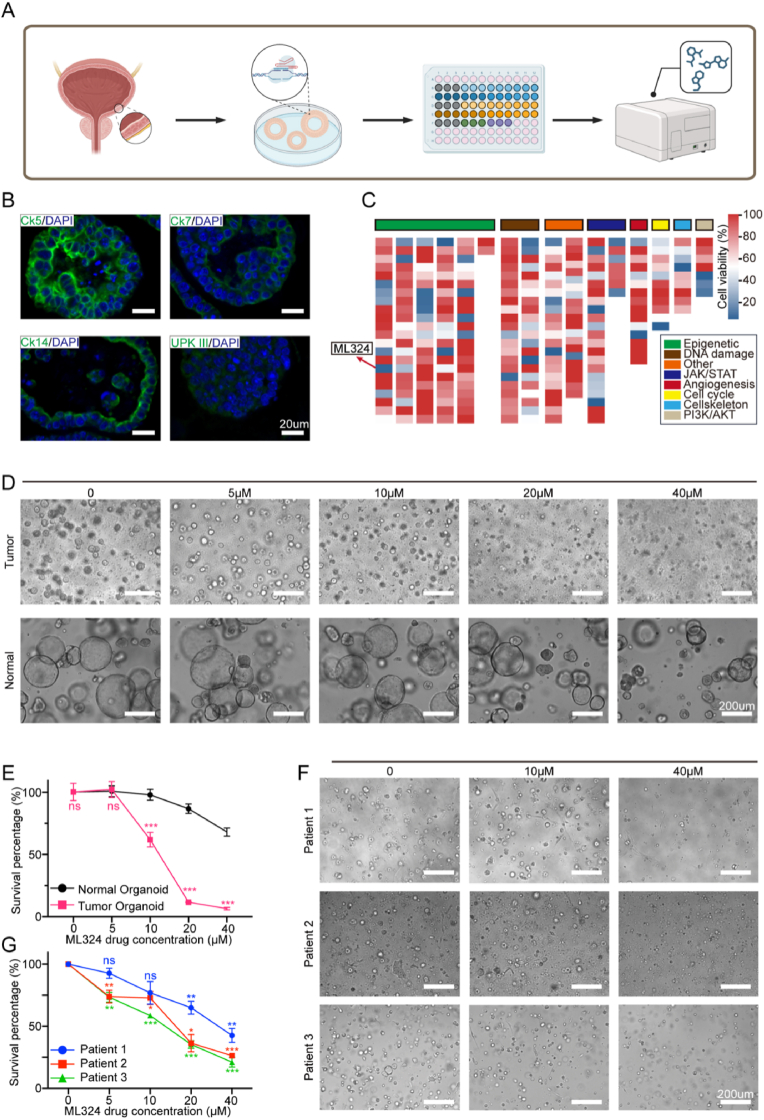
Drug screening identified the antitumor effect of ML324 in bladder cancer organoids. A. Flow chart of bladder cancer organoid construction and drug screening. B. Representative staining of mouse bladder cancer organoids. C. Cell viability of bladder cancer organoids after treatment with molecular inhibitors (at 10 μM) for 72h. Red arrow highlights candidate drug ML324. D, E. Representative images of mouse bladder cancer organoids or normal epithelial organoids treated with vehicle or indicated concentrations of ML324 (D) and viability line chart (E). F, G. Representative images of 3 cases of clinical bladder cancer organoids treated with vehicle or indicated concentrations of ML324 (F) and viability line chart (G).

As reported in our previously published studies [20,21], we established an in vitro drug screening platform based on genetically engineered tumor organoids derived from a primary and orthotopic mouse model of MIBC to evaluate potential antitumor drugs (Fig. 1A). This model featured genetic modifications, including Trp53 and Pten loss and Myc overexpression (sgTrp53, sgPten, Myc [TPM]), and represented the histological and molecular characteristics observed in human MIBC. The derived tumor organoids maintained the pathologic features and expressed the signature of human bladder cancer (Fig. 1B). A total of 276 compounds targeting epigenetic factors were screened. Effects of different compounds on viabilities of tumor organoids were analyzed by CCK8 (drug concentration: 10 μM). Of which #114 drug (red arrow marked), named ML324, inhibited the growth of bladder cancer organoids by 71.6 %, showing the most potent inhibitory effect among histone demethylase inhibitors (Fig. 1C).

### 3.2 ML324 inhibits MIBC tumor growth

To validate the inhibitory effect of ML324 on tumor growth in vitro, varied concentrations of ML324 were applied to treat tumor organoids and the cell viability were evaluated subsequently by CCK8. The results showed, as the drug concentration increased, the proportion of living organoids gradually decreased and reached nearly 100 % growth inhibition at a concentration of 20 μM. On the contrary, the impact on cell growth was modest with ML324 in the lower concentration range (5–20 μM), and approximately 30 % inhibition was noted at the highest concentration of 40 μM in normal bladder epithelial organoids (Fig. 1D and E). Furthermore, three human MIBC organoids were established from patients who underwent radical cystectomy to evaluate the suppression activity of ML324. The results demonstrated that ML324 inhibited the human MIBC organoids growth by a dose-dependent manner, with estimated IC50 around 10 μM (Fig. 1F and G).

Through in vitro toxicity assessments, we have confirmed that ML324 exhibits significant inhibitory effects on both mouse and human bladder cancer organoids. At the same time, this compound demonstrates a favorable safety profile for normal bladder epithelium organoids.

### 3.3 Kdm4a as a druggable target to eradicate bladder cancer organoid with ML324

**Fig. 2:**
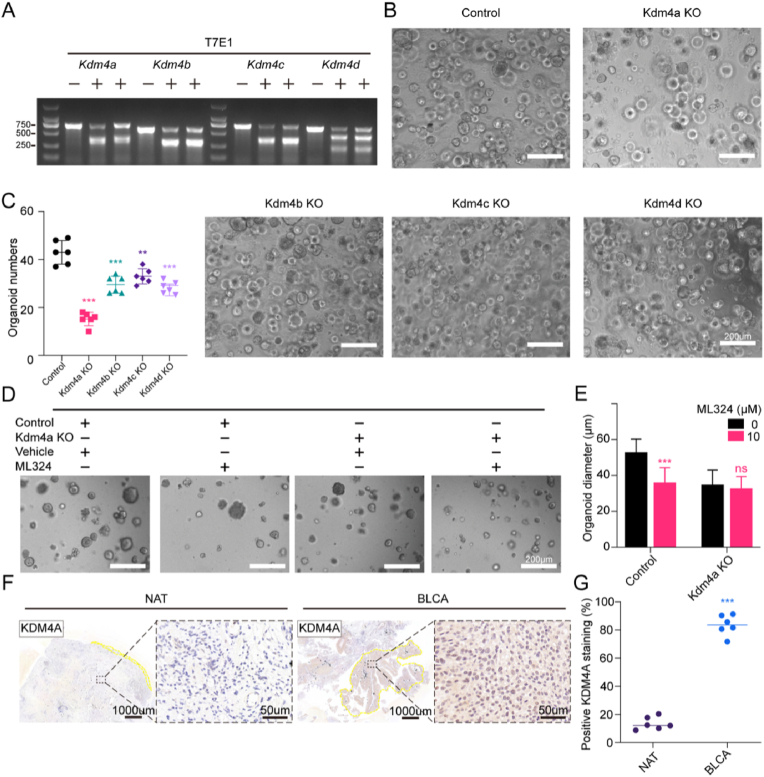
Kdm4a as a druggable target to eradicate bladder cancer organoids with ML324 A. Detection of sgRNA-guided genome editing by T7E1 assays on Kdm4a-d sgRNAs (“+” marked representing 2 distinct sgRNAs) compared to Scramble (“-” marked). B. Representative images of bladder cancer organoids in control or Kdm4a-d knockout groups. C. Relative changes in organoid formation ability in control or Kdm4a-d knockout groups. D. Representative images of ML324 intervention in bladder cancer organoids with or without Kdm4a knockout. E. Relative changes in organoid size in control or Kdm4a-d knockout groups. F. Representative images of KDM4A staining from bladder cancer tissue or normal tissues adjacent to the tumor (NAT). G. Proportion of cells staining positive for KDM4A from BLCA or NAT.

We have preliminarily identified ML324, discovered as a selective histone demethylase JMJD2/KDM4 inhibitor, has a notable inhibitory impact on the growth of bladder cancer organoids in both mouse and human models, showing superior efficacy compared to other KDM4 inhibitors (IOX1 and JIB-04, sFig. 1A and 1B). The roles of KDM4 family members, including KDM4A, KDM4B, KDM4C and KDM4D, on bladder cancer remains unclear. Thus, to clarify the role of KDM4 on the growth inhibition of bladder cancer organoids, control and knockout groups were established by transducing organoids with lentiviral vectors expressing either non-targeting control (scramble) or sgRNAs targeting Kdm4a/b/c/d, respectively. The presence of mutations of KDM4 were confirmed by T7E1 (Fig. 2A). Results showed that mutations of all KDM4 family members significantly reduced the numbers of tumor organoids compared with control group. Notably, mutations in Kdm4a exhibit the most pronounced inhibitory effects on the growth of cancer organoids among them (Fig. 2B and C).

To investigate whether the tumor suppressing effect of ML324 depends on the normal function of Kdm4a, we assessed the impact of ML324 (10 μM) on growth of tumor organoids in Kdm4a knockout or control group. The results indicate consistency with above findings, demonstrating that ML324 exhibits a significant inhibitory effect on bladder tumor organoids. However, the inhibitory effect of ML324 is markedly reduced upon Kdm4a disruption (Fig. 2D and E). Furthermore, an analysis was conducted on the immunohistochemical (IHC) staining of KDM4A in tumor tissue of human MIBC and paired normal bladder tissues from six patients. The findings revealed predominant expression of KDM4A in the nucleus, with a significantly elevated expression level in tumor tissues in comparison to normal bladder tissues (Fig. 2F and G). Our preliminary discoveries suggest a noteworthy role of Kdm4a in sustaining the growth of bladder tumor organoids within the Kdm4 family. Essentially, ML324 could impede the growth of bladder cancer by targeting Kdm4a.

### 3.4 ML324 suppressed the growth of orthotopic bladder cancer in mice

**Fig. 3:**
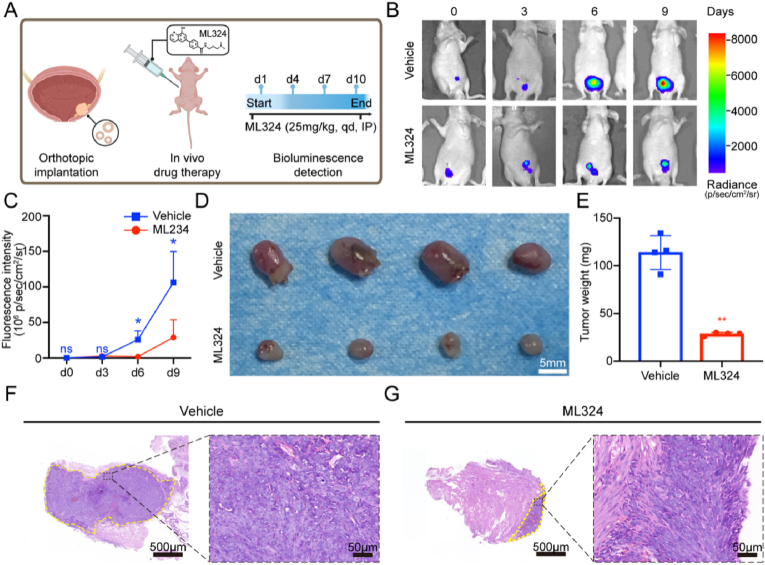
ML324 suppressed the growth of orthotopic bladder cancer in mice A. Scheme showing orthotopic implantation of bladder cancer organoids followed by ML324 treatment and bioluminescent detection. B, C. Bioluminescent images of bladder xenografts derived from mouse bladder cancer organoids in mice treated with vehicle or 25 mg/kg ML324 (B) and fluorescence intensity changes (C). D. Representative images of bladder xenografts harvested from mice treated with vehicle or ML324 (25 mg/kg) for 9 days. E. Bladder xenografts weight after treatment (Mean ± SD, n = 4 replicates per group, ∗∗p < 0.01). F, G. Hematoxylin and eosin staining for bladder xenografts in mice treated with vehicle (F) or ML324(25 mg/kg) (G).

To assess the impact of the ML324 drug on tumor growth in vivo, furtherly, orthotopic bladder cancer model was constructed by injecting cultured mouse bladder cancer organoids into nude mouse bladder wall after digestion, and the tumor volumes were measured by bioluminescence images (Fig. 3A). Five days after transplantation, tumors with comparable sizes were randomly allocated to vehicle group or the ML324 group (25 mg/kg, I.P., qd). Following a 9-day treatment period, the administration of ML324 led to a notable inhibition of bladder tumor growth, as evidenced by fluorescence intensity monitoring (Fig. 3B and C). Upon sacrificing mice at end of the treatment, the administration of ML324 demonstrated a marked decrease in both size and weight of orthotopic bladder tumors, as compared to the vehicle group. (Fig. 3D and E). Following H&E staining of bladder tissue sections, tumors in the vehicle group exhibited exophytic growth characteristics, whereas those in the treatment group displayed a diminished infiltration of tumor cells into the bladder muscle layer (Fig. 3F and G). These findings underscored the efficacy of ML324 in suppressing bladder tumors in vivo.

### 3.5 KDM4A knockout affects the transcriptional landscape of bladder cancer organoids

**Fig. 4:**
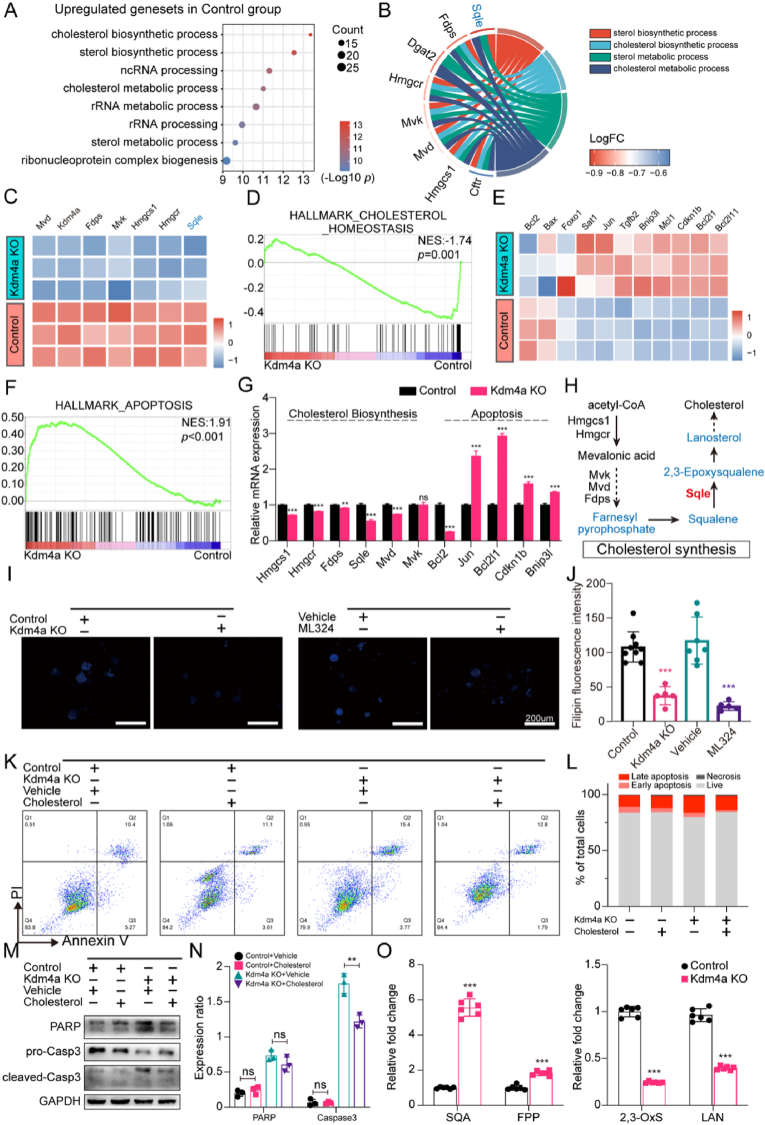
Transcriptional profiling of bladder cancer organoids following Kdm4a Knockout A. Dot plot of the most enriched biological process GO terms upregulated in TPM cells of control group, compared to Kdm4a knockout group. B. GO chord diagram representing the relationship between DEGs and their associated GO terms related to cholesterol/sterol metabolism remodeling. C. Heatmap showing key DEGs involved in cholesterol synthesis before and after KDM4A KO in TPM cells. D. Gene set enrichment analysis (GSEA) showing enrichment profile of CHOLESTEROL HOMEOSTASIS set in TPM cells with or without Kdm4a knockout. E. Heatmap showing key DEGs related to apoptosis before and after Kdm4a knockout in TPM cells. F. Gene set enrichment analysis (GSEA) showing enrichment profile of APOPTOSIS set in TPM cells with or without Kdm4a knockout. G. qRT-PCR analysis of cholesterol synthesis and apoptosis related genes in TPM cells of control group and Kdm4a knockout group. H. Schematic diagram illustrating key genes involved in cholesterol synthesis. I. Representative images of Filipin III staining showing content of cholesterol (blue) in TPM cells inhibited Kdm4a by Kdm4a knockout or ML324 (10 μM), comparing with control or vehicle group (Scale bar 200 μm). J. Fluorescence intensity of Filipin staining in TPM cells of groups in Fig. 4I. K, L. Flow cytometric analysis of apoptosis in response to exogenous cholesterol of TPM cells (control vs Kdm4a KO). M, N. Immunoblots and quantitative analysis for apoptosis markers (cleaved-PARP, cleaved-Caspase3) of groups in Fig. 4K. O. Quantitative analysis of Sqle-regulated metabolite levels in TPM cells (control vs Kdm4a knockout), performed by LC-MS.

To elucidate the mechanisms of KDM4A on suppressing bladder cancer. We examined the transcriptional changes in mouse bladder cancer organoids after Kdm4a knockout using transcriptomics (sFig. 2A and 2B). GO enrichment analysis showed that Kdm4a knockout significantly downregulated steroid/cholesterol biosynthesis and metabolic pathways in TPM cells (Fig. 4A), with Sqle showing the most prominent reduction (Fig. 4B and sFig. 2C). Other key cholesterol synthesis genes also showed decreased expression (Fig. 4C), and GSEA indicated disruption of cholesterol homeostasis (Fig. 4D). Additionally, apoptosis pathways were upregulated (Fig. 4E and F), aligning with the observed growth inhibition and morphological changes in Kdm4a-deficient tumor organoids. mRNA quantification confirmed these trends, with Sqle showing the most pronounced decrease (Fig. 4G and H).

In TPM cells, Kdm4a inhibition, through genetic or pharmacological means, reduced intracellular cholesterol levels, highlighting Kdm4a′s role in cholesterol synthesis (Fig. 4I and J). Despite the importance of cholesterol for rapid tumor cell proliferation and membrane function, our results revealed that exogenous cholesterol supplementation did not significantly reduce apoptosis caused by Kdm4a knockout. (Fig. 4K–N), suggesting that the onset of apoptosis following Kdm4a loss may be attributed to other mechanisms.

### 3.6 KDM4A knockout triggers apoptosis and growth inhibition in an SQLE-dependent manner

Given Sqle's role in oxidizing SQA to 2,3-OxS in cholesterol synthesis, we first tested how Sqle expression levels affect cholesterol biosynthesis in TPM cells. To overexpress Sqle, Sqle-expressing recombinant lentivirus or vector were used to transfect TPM cell (sFig. 3A), leading to a marked increase in cholesterol synthesis, storage, and utilization, while Sqle knockout (sFig. 3B and 3C) reversed these effects (sFig. 3D). Metabolite analysis showed that after Kdm4a knockout, SQA/FPP levels significantly increased, while 2,3-OxS/LAN levels decreased (Fig. 4O), consistent with the function of Sqle as an oxidase. This highlights that the remodeling of cholesterol metabolism in the context of Kdm4a inhibition might tie to Sqle's enzymatic activity.

**Fig. 5:**
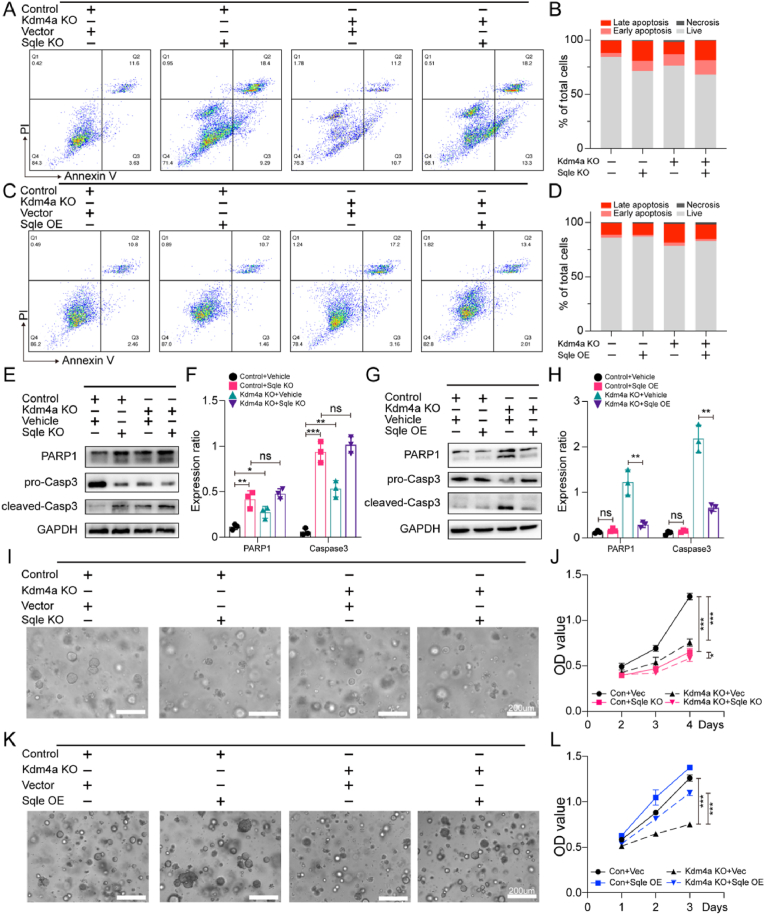
Kdm4a knockout induced apoptosis and growth inhibition in Sqle-dependent manner A, B. Flow cytometric analysis of apoptosis in response to Sqle knockout of TPM cells (control and Kdm4a knockout). C, D. Flow cytometric analysis of apoptosis in response to Sqle overexpression of TPM cells (control and Kdm4a knockout). E, F. Immunoblots and quantitative analysis for apoptosis markers (cleaved-PARP, cleaved-Caspase3) of groups in Fig. 5A. G, H. Immunoblots and quantitative analysis for apoptosis markers (cleaved-PARP, cleaved-Caspase3) of groups in Fig. 5C. I-L. Representative images showing the effects of Sqle knockout (I) or overexpression (K) on the growth and morphology of TPM cells (control or Kdm4a knockout), OD values indicated cell viability via the CCK-8 assay (J, L).

To explore the link between Kdm4a knockout-induced apoptosis and Sqle downregulation, we performed flow cytometry and revealed that apoptosis from Sqle knockout alone was more severe than from Kdm4a knockout (Fig. 5A and B). And the additional knockout of Kdm4a under Sqle-deficient condition resulted in only a slight increase. These data suggest that the pro-proliferative potential of Kdm4a is restricted in the absence of Sqle.

We also test the role of overexpressed Sqle in Kdm4a knockout-induced apoptosis. Consistent with previous findings, Sqle overexpression partially rescued the increased apoptosis in TPM cells with Kdm4a knockout (Fig. 5C and D). Apoptosis markers (cleaved-PARP1 and cleaved-caspase) were upregulated in cells with Kdm4a or Sqle knockout. However, further Kdm4a knockout on the basis of Sqle deficiency did not exacerbate apoptosis (Fig. 5E and F). Conversely, under normal Kdm4a conditions, Sqle overexpression had no significant impact on apoptosis markers, but it significantly reduced apoptosis in Kdm4a knockout cells (Fig. 5G and H). These results suggest that Sqle downregulation is a major driver of apoptosis following Kdm4a knockout, likely through caspase pathway activation.

Similar results were observed in TPM cell proliferation. While both Kdm4a and Sqle loss inhibited tumor organoids growth to varying degrees, with Sqle downregulation having a more pronounced effect, comparable to that of double knockout (Fig. 5I and J). Transwell assays showed that the reduced invasion and migration of TPM cell with Kdm4a knockout were also dependent on Sqle downregulation (sFig. 3E and 3F). Additionally, Sqle overexpression enhanced organoid growth regardless of Kdm4a status, with a more pronounced rescue of the growth inhibition caused by Kdm4a knockout (Fig. 5K and L).

### 3.7 Clearance of ROS by SQA accumulation following the KDM4A-SQLE axis

Our data suggest that Kdm4a inhibition downregulates Sqle, inducing apoptosis through a non-cholesterol-dependent pathway. To explore how the Kdm4a-Sqle axis triggers apoptosis, we focused on the potential role of accumulated SQA. Previous studies have demonstrated SQA's role as an antioxidant by reducing ROS and lipid peroxidation, protecting cells from oxidative damage, though it is generally secondary and evident under high oxidative stress [22]. It remains unclear whether excessive SQA, resulting from Sqle deficiency, would overly enhance its antioxidant function and how this impacts cells.

**Fig. 6:**
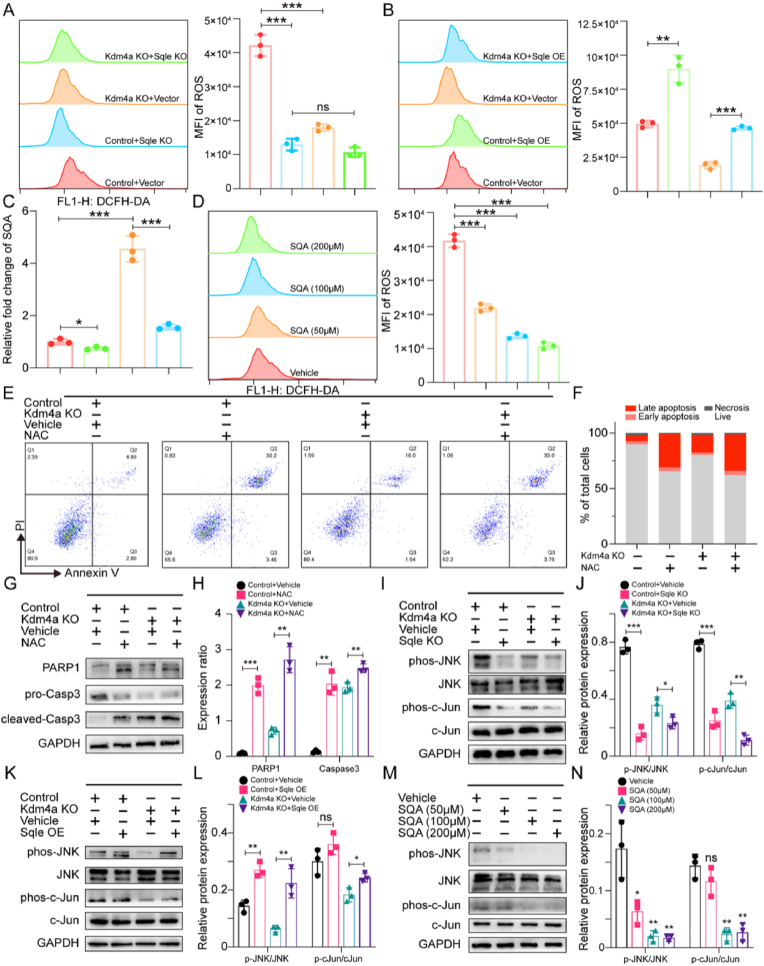
Inhibition of the Kdm4a-Sqle axis induces ROS clearance and JNK/c-Jun deactivation A. Flow cytometric analysis and histograms representing the ROS levels in response to Sqle knockout of TPM cells (control and Kdm4a knockout). B. Flow cytometric analysis and histograms showing the effects of Sqle overexpression on ROS levels in TPM cells (control and Kdm4a knockout). C. Relative intracellular SQA expression level of groups in Fig. 6B (data of control group was normalized to 1). D. Flow cytometric analysis and histograms illustrating the effect of ectopic SQA on ROS clearance in TPM cells. E, F. Flow cytometric analysis of apoptosis in response to ROS clearance (performed by NAC) of TPM cells (control and Kdm4a knockout). G, H. Immunoblots and quantitative analysis for apoptosis markers (cleaved-PARP, cleaved-Caspase3) of groups in Fig. 6E. I, J. Immunoblots and quantitative analysis showing activation level of JNK/cJUN pathway of groups in Fig. 6A. K, L. Immunoblots and quantitative analysis showing activation level of JNK/cJUN pathway of groups in Fig. 6B. M, N. Immunoblots and quantitative analysis showing activation level of JNK/cJUN pathway of groups in Fig. 6D.

As shown in Fig. 6A, both Kdm4a and Sqle knockout significantly reduced ROS levels, the latter decreased ROS to nearly undetectable levels. Therefore, additional Kdm4a knockout in the context of Sqle deficient did not further reduce ROS level. Sqle overexpression reversed this effect, restoring intracellular ROS levels in the context of Kdm4a knockout (Fig. 6B), as well as SQA levels to near-normal (Fig. 6C).

To confirm that SQA is responsible for the ROS reduction, we treated TPM cells with varying concentrations of exogenous SQA under normal Kdm4a expression. As shown in Fig. 6D, ROS levels decreased in a dose-dependent manner, suggesting that accumulated SQA contributes to ROS clearance.

To link ROS reduction with apoptosis, we treated Kdm4a knockout TPM cells with the ROS scavenger NAC. Flow cytometry analysis revealed that NAC-induced apoptosis, but additional Kdm4a knockout did not further increase the apoptosis rate (Fig. 6E and F). Detection of protein level of cleaved-PARP1 and cleaved-caspase3 confirmed that NAC-mediated ROS clearance triggered apoptosis, and further ROS removal in the context of Kdm4a knockout did not significantly exacerbate the level of apoptosis (Fig. 6G and H). Thus, we propose that the disruption of oxidative stress balance caused by Kdm4a inhibition is a key driver of apoptosis in TPM cells.

### 3.8 KDM4A-SQLE-SQA axis affects the activation of the ROS/JNK/c-Jun pathway

ROS in tumor cells can either promote tumor progression or induce apoptosis depending on its levels [23]. At low levels, ROS primarily activates the JNK/c-Jun pathway, which functioned to drive tumor progression. We investigated whether ROS clearance due to Kdm4a inhibition affects the ROS/JNK/c-Jun pathway through a similar mechanism.

Western blot analysis showed that reduced phosphorylation levels of JNK and c-Jun suggest decreased activation of the JNK/c-Jun pathway following Kdm4a knockout. The reduction in phosphorylation caused by Sqle knockout was more pronounced, while the JNK/c-Jun pathway activation level in the double knockout group was comparable to that of Sqle knockout alone (Fig. 6I and J). To determine whether such modulation depends on Sqle and its regulation of SQA, we observed that Sqle overexpression worked to activate JNK/c-Jun phosphorylation and restored the pathway to near control levels in Kdm4a knockout cells (Fig. 6K and L). Conversely, the addition of exogenous SQA effectively reduced JNK/c-Jun phosphorylation in TPM cells under normal Kdm4a level (Fig. 6M and N). These findings confirm our hypothesis that Kdm4a knockout disrupts the tumor-promoting function of ROS through JNK/c-Jun activation. Phosphorylated c-Jun was reported to upregulate anti-apoptotic genes and inhibiting apoptosis [24,25], which aligns with our transcriptional data showing changes in Bcl2 family members (Bax, Bcl2) following Kdm4a knockout (Fig. 4E). This potentially explains part of the mechanism underlying apoptosis in Kdm4a-deficient cells.

### 3.9 KDM4A activates SQLE expression via H3K9me3 demethylation, enabling GABPA binding to the promoter

**Fig. 7:**
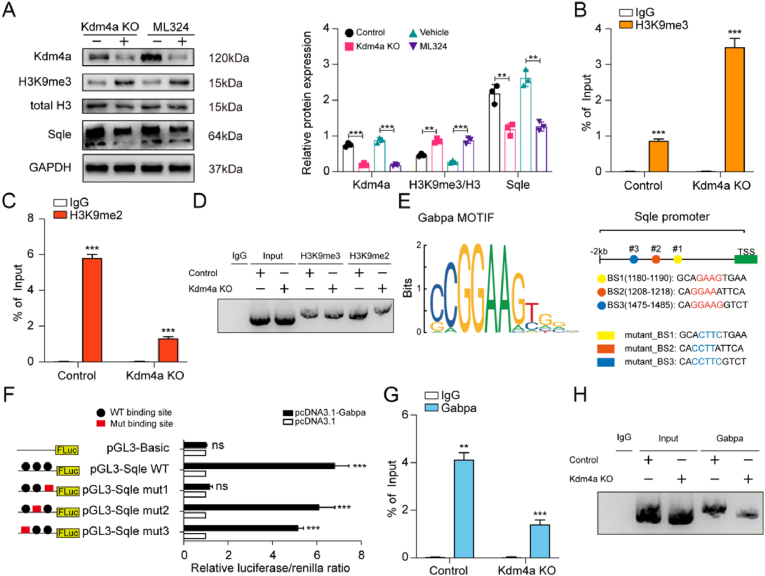
Kdm4a regulates Gabpa-mediated transcriptional activation of Sqle A. Immunoblots for Kdm4a, H3K9me3 and Sqle level in TPM cells with or without Kdm4a knockout. B, C. ChIP-qPCR for the binding of H3K9me3 (B) and H3K9me2 (C) at the Sqle promoter region in TPM cells (control vs Kdm4a knockout), respectively. D. Kdm4a affected the binding of H3K9 with different methylation modifications to Sqle promoter region by ChIP-PCR.E. Basing on Gabpa motif, potential binding sites in Sqle promoter region were predicted and relative mutations were constructed in highly conversed bases.F. Luciferase reporter assay identified the direct Gabpa binding site (BS1) in Sqle promoter region by sequential mutation.G. ChIP-qPCR for Gabpa binding at the Sqle promoter region in TPM cells (control vs Kdm4a knockout).H. Kdm4a knockout attenuated Gabpa binding in the Sqle promoter region by ChIP-PCR.

To uncover the molecular mechanism by which Kdm4a regulates Sqle expression, we first assessed Sqle protein levels and observed a significant decrease in TPM cells with Kdm4a inhibition, also a reduction in H3K9 trimethylation levels, which served as a demethylation site for Kdm4a (Fig. 7A).

As a histone mark, H3K9me3 is associated with chromatin compaction and transcriptional repression. Its demethylation to H3K9me2, which is more permissive for transcription, can increase chromatin accessibility. To identify whether Kdm4a-mediated chromatin opening increases accessibility in the Sqle promoter region, we examined the binding of different H3K9 methylation marks to the Sqle promoter by ChIP assays. As expected, H3K9me2 was more enriched at the Sqle promoter in control group (with normal Kdm4a expression) compared to H3K9me3. Following Kdm4a knockout, the opposite was observed, with reduced H3K9me2 enrichment in Sqle promoter region (Fig. 7B and C). Consistent results were seen in the ChIP-PCR assay (Fig. 7D), suggesting that under Kdm4a's influence, the active histone mark H3K9me2 may mediate Sqle transcriptional activation.

To identify transcription factors (TFs) directly involved in Sqle transcription within an open chromatin context, multiple databases and tools (JASPAR, ENCODE, KnockTF, hTFtarget) were used and pointed Gabpa as the top candidate. To validate this, we located 3 potential GABPA binding sites ∼2000bp upstream of the Sqle transcription start site (TSS). Luciferase reporters were constructed with wild type or different mutations in binding sites (Fig. 7E). Mutation of binding site 1 significantly reduced luciferase activity, while mutations in sites 2 and 3 had no effect (Fig. 7F), indicating the critical role of binding site 1 in Gabpa-mediated Sqle transcriptional activation. Considering the role of Kdm4a in chromatin accessibility, ChIP assay was performed to further investigated whether Gabpa's activation of Sqle transcription is dependent on Kdm4a. We observed reduced Sqle promoter region enrichment following Kdm4a knockout (Fig. 7G and H). These findings demonstrated that Kdm4a demethylates H3K9me3, allowing Gabpa to bind directly to the Sqle promoter (1180–1190 region), which is crucial for Sqle transcriptional activation.

### 3.10 ML324 inhibited tumor growth in the human MIBC xenografts

**Fig. 8:**
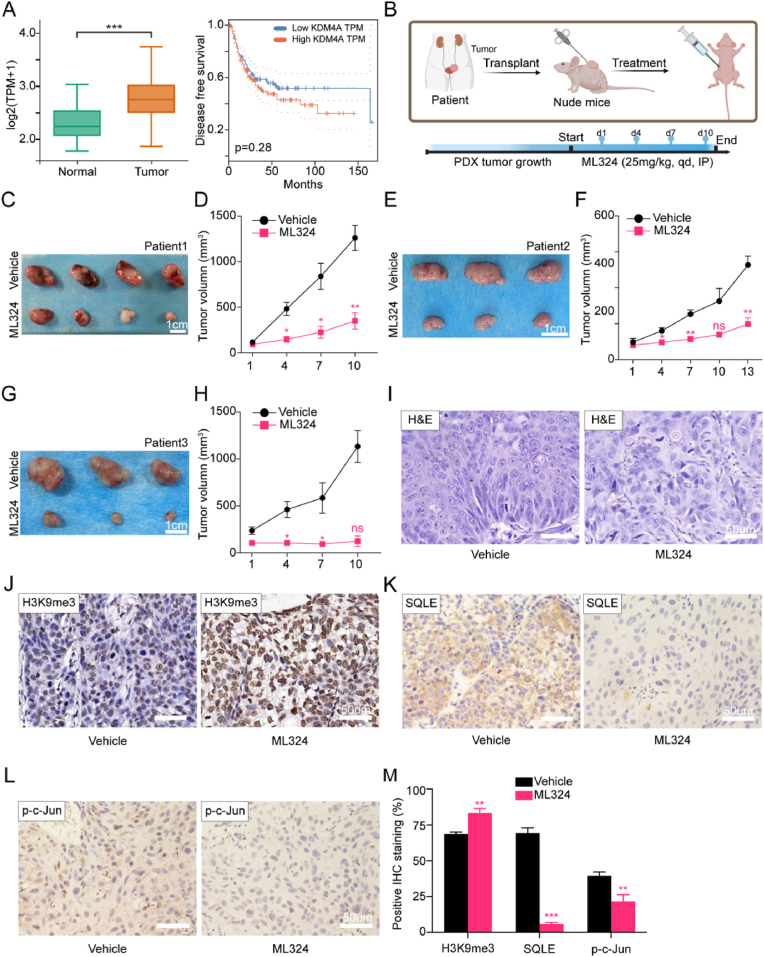
ML324 inhibited tumor growth in the human MIBC xenografts A. TCGA-BLCA cohort showing relative KDM4A expression level on normal or tumor tissue. Kaplan–Meier plots demonstrating the impact of KDM4A levels on BLCA patients' disease-free survival (DFS). B. Scheme illustrating the construction of nude mouse PDX model of BLCA patient tumor specimens and in vivo treatment with ML324 (25 mg/kg). C–H. Representative images of harvested xenografts after treatment of vehicle or ML324 in PDX models constructed from 3 MIBC patients (C, E, G), and tumor volume changes in different groups (D, F, H).I: Hematoxylin and eosin staining in representative bladder xenografts with or without in vivo treatment of ML324.J–M: Immunohistochemical staining for H3K9me3 (J), SQLE (K) and p-*c*-Jun (L), and proportion of positive staining cells (M) in representative bladder xenografts treat with or without ML324 (25 mg/kg) (Scale bar 50 μm).

By analyzing the BLCA_TCGA database, the results suggested that the expression level of KDM4A in bladder cancer was significantly higher than that in normal bladder epithelial tissues (p < 0.001), and patients with higher expression of KDM4A generally had a poor disease-free survival time compared with those with lower KDM4A expression, although the difference was not statistically significant (p = 0.28) (Fig. 8A).

Further, PDX models from 3 MIBC patients have been established and applied to evaluate the therapeutic effect of ML324 on human bladder cancer in vivo (Fig. 8B). Treatment was performed daily by intraperitoneal injection of ML324 (25 mg/kg) or vehicle, and subcutaneous tumor size were measured every three days. Upon completion of the 14-day course or tumor size approaching 1500 mm^3^, subcutaneous xenografts were harvested. The results showed the tumor growths were significantly suppressed by ML324 treatment compared to the vehicle group in all three PDX lines (Fig. 8C–H).

The inhibition of Kdm4a, resulting in the downregulation of Sqle and subsequent suppression of ROS/JNK/c-Jun signaling pathway, has been clearly elucidated in mouse bladder cancer models as described above. We further examined the expression levels of key proteins of the Kdm4a-Sqle-JNK/c-Jun axis by IHC staining in human MIBC xenografts treated with ML324. Consistent with mouse orthotopic models, ML324 treatment led to a significant increase in trimethylation levels at the H3K9 locus, accompanied by a downregulation of SQLE expression and a decrease in the p-*c*-Jun in human bladder cancer (Fig. 8I–M). These findings demonstrated that ML324 could also suppress the growth of human bladder cancer in vivo. Moreover, the KDM4A-SQLE-JNK/c-Jun axis, which could be disrupted by ML324, played a crucial role in human MIBC.

## 4 Discussion

In this study, we observed transcriptional changes following Kdm4a knockout in bladder cancer organoids, primarily characterized by impaired cholesterol synthesis and increased apoptosis (Fig. 4). Metabolic reprogramming is well-recognized as an adaptive change in tumor cells to meet the energy and metabolic demands of rapid proliferation [26]. Enhanced cholesterol synthesis may provide essential materials for the formation of new cell membranes in rapidly dividing cells or contribute to tumor survival through intermediate metabolites that promote proliferation and offer antioxidant protection [13].

Previous studies have reported the broad impact of cholesterol deficiency on cell and organelle membranes [27,28]. In our study, we first assessed the importance of cholesterol insufficiency in maintaining the viability of tumor organoids. Notably, supplementing tumor organoids with exogenous cholesterol provided only limited rescue from apoptosis. This may be explained by the short-term compensatory mechanisms, such as redistribution of intracellular cholesterol pools or increased uptake of exogenous cholesterol [29], which partially mitigates the impact of synthesis deficiency. However, it also suggests that apoptosis likely stems from earlier and more severe disruptions in intracellular homeostasis.

Further bioinformatics analysis highlighted Sqle as the most significantly altered gene in the cholesterol synthesis pathway. As the first oxidase in this process, Sqle catalyzes an essential, non-redundant step in the oxidation of SQA. We then investigated whether Sqle, as an upstream regulator of cholesterol synthesis, is responsible for apoptosis. Interestingly, the results showed that Kdm4a knockout-induced growth inhibition and apoptosis in tumor organoids are highly dependent on Sqle (Fig. 5). From the perspective of Sqle's enzymatic function, we further questioned whether its upstream product, SQA, might be the critical factor influencing cell survival.

SQA, beyond being an intermediate in cholesterol synthesis, caught our attention due to its antioxidant properties. Tumor cells are characterized by heightened oxidative stress, mainly reflected by increased ROS levels. It is widely accepted that ROS plays a dual role in the tumor microenvironment, promoting proliferation and survival at low levels but inducing cell damage and apoptosis at high levels [23]. When SQA accumulates due to insufficient Sqle expression, it may rapidly eliminate ROS, thereby inhibiting the growth advantage driven by low ROS levels. Our results support this hypothesis as Kdm4a knockout reduced ROS levels in tumor cells, and similar ROS clearance could be induced by exogenous SQA supplementation. Sqle overexpression partially restored ROS levels caused by Kdm4a knockout, resulting in partially recovery of cell growth and reduced apoptosis. To identify whether Kdm4a knockout-induced apoptosis depends on ROS clearance, we used ROS scavengers and found that additional Kdm4a knockout did not further increase apoptosis. However, maintaining ROS at low levels to promote cell growth via exogenous H_2_O_2_ proved challenging, as excessive H_2_O_2_ tends to cause excessive ROS accumulation and oxidative damage. Thus, we were unable to fully confirm the dependency of apoptosis on ROS through the supplementation strategy in the context of Kdm4a deficiency.

The relationship between KDM4A and hypoxic characteristics may also merit further consideration, particularly in the context of KDM4A's role in regulating cell survival through its influence on oxidative stress balance. Notably, Martinez et al. reported that, in tumor spheroids, outer tumor cells exhibit enhanced KDM4A expression and activity due to intermittent hypoxia, leading to the activation of the HIF pathway. Conversely, chronic hypoxia was found to reduce KDM4A-C activity in the inner tumor cells [30]. This phenomenon may significantly contribute to the heterogeneous response of different tumor layers to KDM4A-targeted therapies in vivo.

Although ROS regulates numerous downstream pathways, the JNK/c-Jun signaling pathway is one of the key routes activated under low ROS levels to promote tumor growth [[31], [32], [33]]. Research in schwannomas has indicated that activated JNK can protect cells from apoptosis by limiting further accumulation of ROS [34]. This finding is intriguing as it aligns with our observation in this study, where moderate levels of ROS in tumor cells during KDM4A expression may exert a pro-survival effect. Furthermore, it partially elucidates the dual role that JNK may play in cell survival [35]. Once phosphorylated, JNK/c-Jun can regulate a wide array of genes. Our data show that JNK/c-Jun activation was significantly higher in the control group with normal Kdm4a expression, which may provide a growth advantage for the tumor organoids. In contrast, Kdm4a knockout drastically reduced JNK/c-Jun phosphorylation.

While the precise molecular mechanisms by which reduced JNK/c-Jun activation leads to apoptosis were not extensively explored in this study, it likely involves multiple genes. In addition to its potential effects on anti-apoptotic pathways such as the Bcl2 family, c-Jun's role in cell cycle regulation is also noteworthy. c-Jun has been reported to accelerate the cell cycle via CCND1 [36], while its opposing protein, CDKN1B, was transcriptionally upregulated following Kdm4a knockout in our data. This suggests a possible mechanism by which insufficient c-Jun activation may lead to cell cycle inhibition and, ultimately, apoptosis.

Although our study demonstrates that Sqle transcription is more influenced by chromatin structure than by Gabpa levels, and that the KDM4A/GABPA/SQLE axis is highly conserved, species-specific differences in chromatin accessibility should be considered when extending the GABPA-SQLE mechanism to human BLCA. Moreover, we found a moderate correlation between GABPA and SQLE expression levels in the TCGA_BLCA dataset (sFig. 4), which suggests that validating whether the KDM4A-GABPA-SQLE regulatory mechanism is conserved in human BLCA models, both in vitro and in vivo, remains essential.

Our findings highlighted Sqle's critical role in mediating the antitumor effect of Kdm4a inhibition in bladder cancer. Previous studies have linked Sqle to bladder cancer growth via p53-mediated signaling pathways, exploring its potential as a therapeutic target [37]. However, the connection between Sqle's oncogenic role and its metabolic regulatory functions has not been fully explored. Recently, there has been growing interest focused on tumor metabolism. While therapies targeting metabolic enzymes offer direct intervention, metabolic redundancy can lead to rapid resistance [38,39]. Tumor progression is driven by a complex interplay of biological processes, as seen in our study, where the relationship between the metabolic byproduct SQA and oxidative stress balance was evident. Targeting the upstream regulator KDM4A could simultaneously affect multiple pathways, including cholesterol synthesis, offering a broader inhibition of tumor growth. That said, the potential for increased side effects must also be considered. Therapeutic agents must achieve high specificity, and ideally, tumor-selectivity, to avoid unnecessary toxicity.

Overall, we identified the KDM4A inhibitor ML324, which demonstrated a potent cytotoxic effect on both human and mouse bladder cancer models. Mechanistically, we propose that KDM4A inhibition alters chromatin structure, preventing GABPA from activating SQLE transcription. This leads to the accumulation of SQA, which scavenges ROS and downregulates JNK/c-Jun activation, ultimately inducing apoptosis in bladder cancer cells. In summary, our findings suggest that KDM4A, as a druggable epigenetic regulator, holds potential as a novel target for assessing malignancy risk and developing new therapeutic strategies for bladder cancer.

## CRediT authorship contribution statement

**Jiapeng Zhang:** Writing – review & editing, Writing – original draft, Visualization, Validation, Investigation. **Hang Xu:** Writing – original draft, Formal analysis. **Yirui He:** Methodology, Conceptualization. **Xiaonan Zheng:** Data curation. **Tianhai Lin:** Resources. **Lu Yang:** Supervision, Resources. **Ping Tan:** Conceptualization. **Qiang Wei:** Supervision, Resources, Funding acquisition.

## Declaration of generative AI and AI-assisted technologies in the writing process

During the preparation of this work the author(s) used ChatGPT in order to assist with refinement. After using this tool/service, the author(s) reviewed and edited the content as needed and take(s) full responsibility for the content of the publication.

## Funding statement

This study was supported by Postdoctoral Fellowship Program of CPSF (grant number: GZC20231829), 1.3.5 project for disciplines of excellence, West China Hospital, Sichuan University ZYGD23001, the National Natural Science Foundation of China (82473411), and Sichuan Science and Technology Program (2023NSFSC1905).

## Declaration of competing interest

The authors declare no conflict of interest.

## Data Availability

All the data supporting the findings of this study are available within the article and its supplemental files.
