# Supplementary material for: Inhibition of KDM4A restricts SQLE transcription and induces oxidative stress imbalance to suppress bladder cancer
Source: Redox Biol. 2024 Oct 22;77:103407. doi: 10.1016/j.redox.2024.103407 (PMC11543538; doi:10.1016/j.redox.2024.103407)
Supplement: Multimedia component 1 [file mmc1.docx]

**
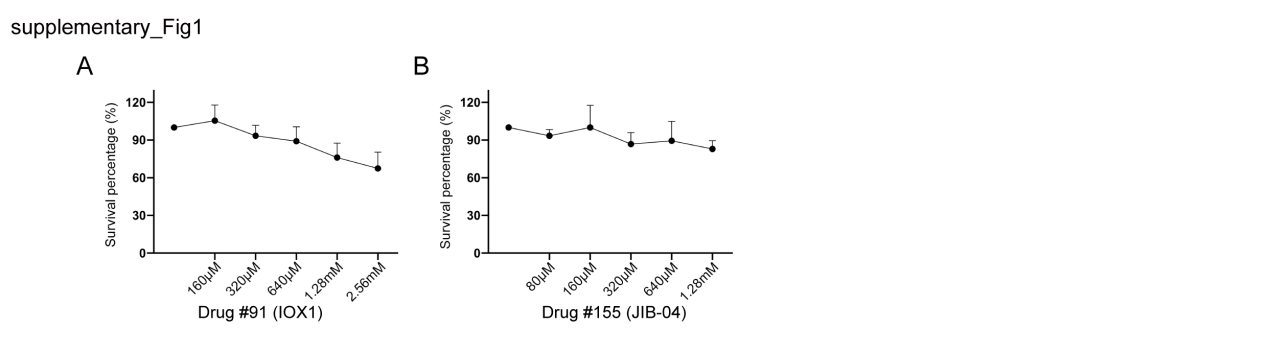
**

**Supplementary Figure 1. Inhibition efficacy of KDM4 inhibitors on bladder cancer organoids**

1. Effects of increasing IOX1 concentration on the survival percentage of bladder cancer organoids.
2. Effects of increasing JIB-04 concentration on the survival percentage of bladder cancer organoids. Data are presented as mean ± SD from three independent experiments.

**
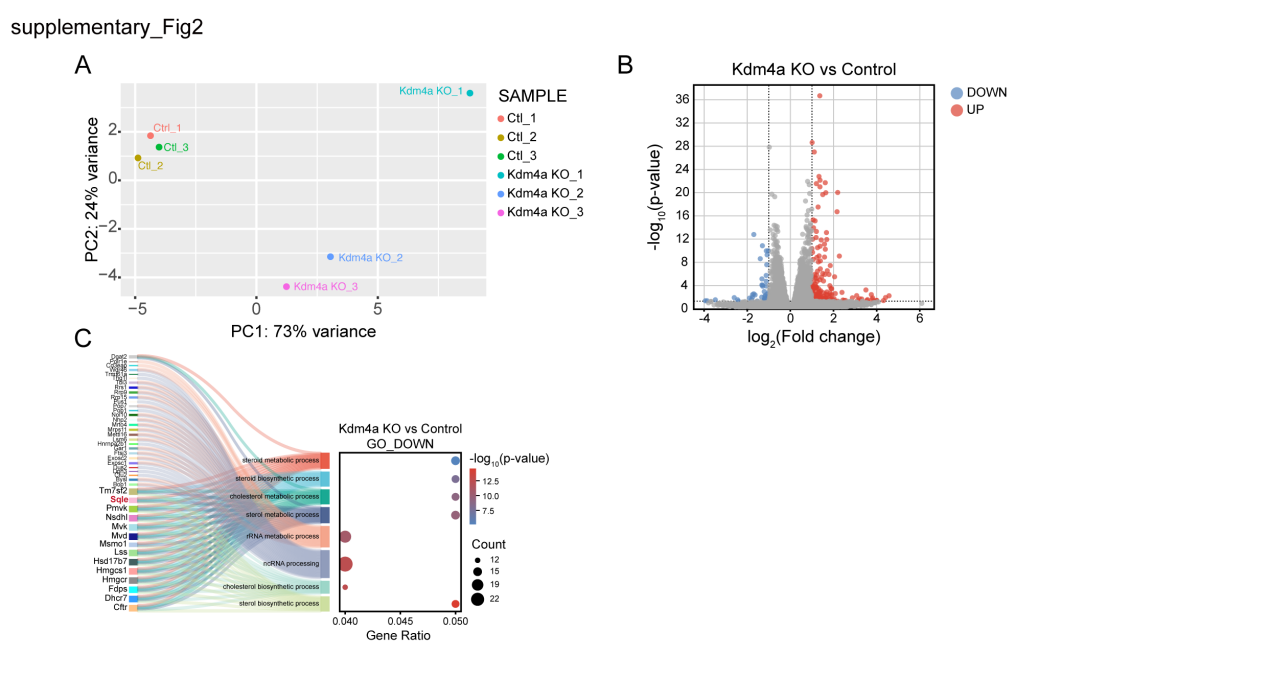
**

**Supplementary Figure 2. Quality control and bioinformatic analysis of transcriptomic sequencing following Kdm4a knockout**

A. Principal component analysis (PCA) showing of gene expression signature obtained from RNA-sequencing for 3 biological replicates from TPM cells with or without Kdm4a knockout.

B. Volcano plot of gene expression levels from all samples, genes were labelled as UP or DOWN regulated with fold change of expression greater than 1.

C. Sankey plot showing DEGs specific to down-regulated GO terms of biological process between TPM cells with Kdm4a knockout and control.


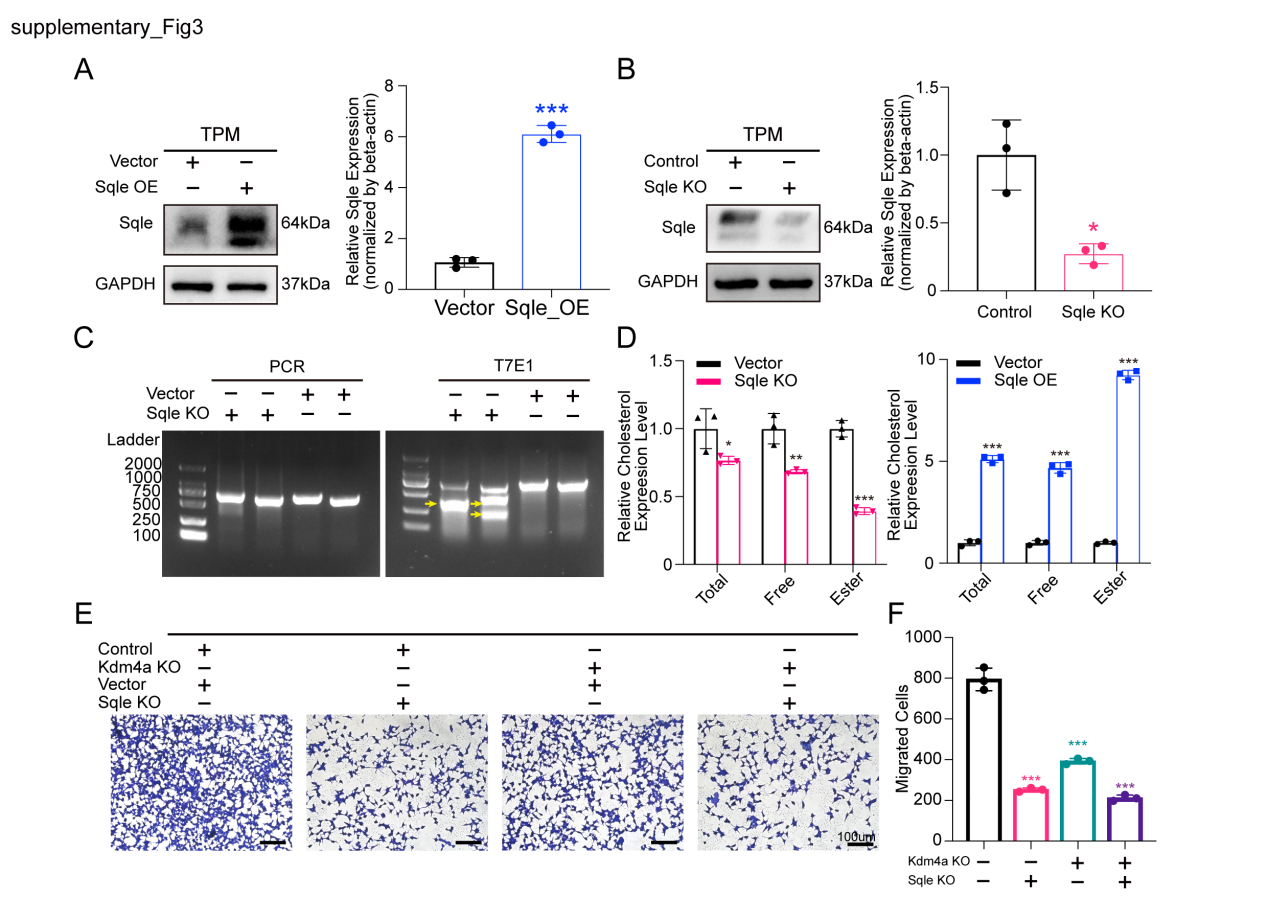


**Supplementary Figure 3. Identification of Sqle expression intervention and the its effects on cholesterol utilization and cell invasioness.**

A. Western blot and semi-quantitative analysis of Sqle expression levels in TPM cells transduced with Sqle-overexpressing lentiviral or vector control.

B. Western blotting and semi-quantitative analysis of Sqle expression levels in TPM cells with or without Sqle knockout.

C. Detection of sgRNA-guided genome editing by T7E1 assays on Sqle-sgRNAs compared to the control.

D. Quantitative analysis of cholesterol synthesis and utilization levels in TPM cells following knockout or overexpression of Sqle, performed by LC-MS.

E, F. Representative images (E) and quantitative analysis (F) of transwell assay of TPM cells in the presence of Kdm4a/Sqle knockout alone or in combination, respectively.


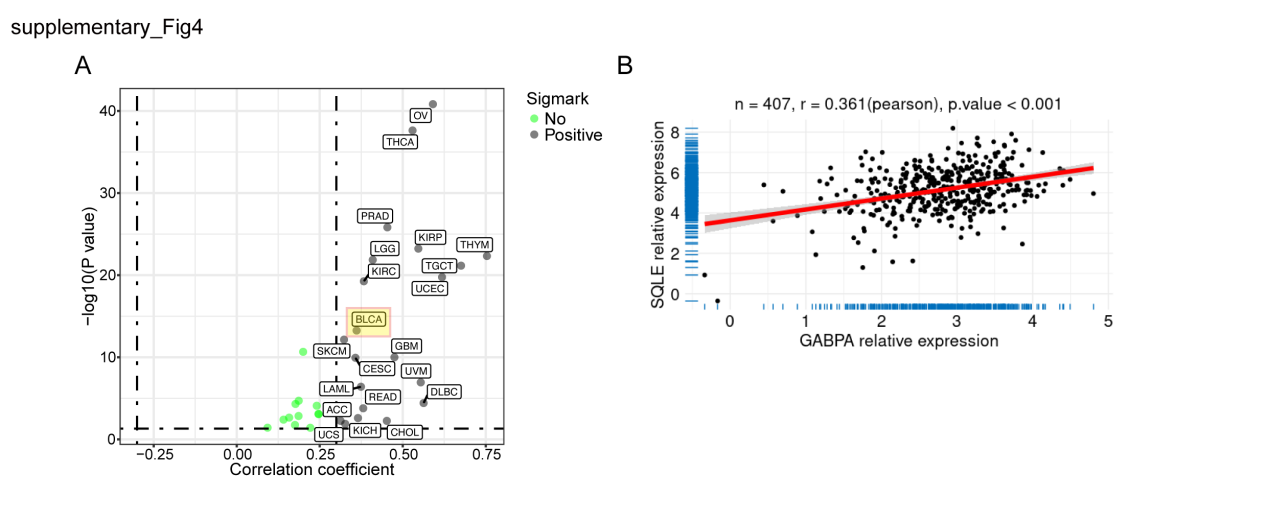


**Supplementary Figure 4. Correlation between Gabpa and Sqle expression in tumors**

A. Correlation of Gabpa and Sqle in pan-cancer.

B. Correlation of Gabpa and Sqle expression in bladder cancer, data collected from TCGA_BLCA cohort.

Supplementary Table 1: **Antibodies used in this study**

| Antibody | Company | Catalog Number | Application &Dilutions |
| --- | --- | --- | --- |
| KDM4A | Abcam | ab191433 | WB 1:1000; IHC 1:200 |
| SQLE | Proteintech | 12544-1-AP | WB 1:1000; IHC 1:200 |
| H3K9me3 | Abcam | ab8898 | ChIP 1:20 |
| H3K9me2 | Abcam | ab1220 | ChIP 1:20 |
| Histone3 | Abcam | ab1791 | WB 1:2000 |
| GAPDH | Abcam | ab181602 | WB 1:10000 |
| GABPA | Sigma-Aldrich | ABE1845 | ChIP 1:30 |
| CK5 | RTM BIO | PTM-5040 | IF 1:50 |
| CK7 | Abcam | ab181598 | IF 1:100 |
| CK14 | Abcam | ab192055 | IF 1:100 |
| PARP1 | Abcam | ab32138 | WB 1:2000 |
| Caspase3 | CST | #9662 | WB 1:1000 |
| Cleaved-Caspase3 | CST | #9661 | WB 1:1000 |
| JNK | CST | #9252 | WB 1:1000 |
| phos-JNK | CST | #4668 | WB 1:1000 |
| cJUN | CST | #9165 | WB 1:1000 |
| phos-cJUN | CST | #3270 | WB 1:1000 |

Supplementary Table 2: **Primers used in this study**

| **guide sequence for CRISPR/Cas9** | | |
| --- | --- | --- |
| Target gene | Forward sequence | Reverse sequence |
| Kdm4a sg1 | caccgCAATTTTGCTACCCGTCGG | aaacCCGACGGGTAGCAAAATTGc |
| Kdm4a sg2 | caccgAGATCATCAATATCGTCGT | aaacACGACGATATTGATGATCTc |
| Kdm4b sg1 | caccGGGCACTTACATCGTCGTAC | aaacGTACGACGATGTAAGTGCCC |
| Kdm4b sg2 | caccgGGCACTTACATCGTCGTAC | aaacGTACGACGATGTAAGTGCCc |
| Kdm4c sg1 | caccgAGAGTTGGCGCCATCCACT | aaacAGTGGATGGCGCCAACTCTc |
| Kdm4c sg2 | caccGTCATTGGGGATCTGGCCGG | aaacCCGGCCAGATCCCCAATGAC |
| Kdm4d sg1 | caccgTGGTGCTTTGGTCACCCGC | aaacGCGGGTGACCAAAGCACCAc |
| Kdm4d sg2 | caccgCGTTACTTACCGTGGCTAT | aaacATAGCCACGGTAAGTAACGc |
| Sqle sg1 | caccgGTAATCGGCGTGCAATACA | aaacTGTATTGCACGCCGATTACc |
| Sqle sg2 | caccGAAGAGTCCATCCGCAACAA | aaacTTGTTGCGGATGGACTCTTC |
| **qPCR primers** | | |
| Hmgcs1 | AACTGGTGCAGAAATCTCTAGC | GGTTGAATAGCTCAGAACTAGCC |
| Hmgcr | AGCTTGCCCGAATTGTATGTG | TCTGTTGTGAACCATGTGACTTC |
| Fdps | GGAGGTCCTAGAGTACAATGCC | AAGCCTGGAGCAGTTCTACAC |
| Sqle | ATAAGAAATGCGGGGATGTCAC | ATATCCGAGAAGGCAGCGAAC |
| Mvd | ATGGCCTCAGAAAAGCCTCAG | TGGTCGTTTTTAGCTGGTCCT |
| Mvk | GGTGTGGTCGGAACTTCCC | CCTTGAGCGGGTTGGAGAC |
| Bcl2 | ACGTGGACCTCATGGAGTG | TGTGTATAGCAATCCCAGGCA |
| Jun | CCTTCTACGACGATGCCCTC | GGTTCAAGGTCATGCTCTGTTT |
| Bcl2l1 | GACAAGGAGATGCAGGTATTGG | TCCCGTAGAGATCCACAAAAGT |
| Cdkn1b | TCAAACGTGAGAGTGTCTAACG | CCGGGCCGAAGAGATTTCTG |
| Bnip3l | ATGTCTCACTTAGTCGAGCCG | CTCATGCTGTGCATCCAGGA |
| **Primers for CDS amplification** | | |
| Sqle-F | TGAGCCATGTGGACTTT | |
| Sqle-R | CCCTTTCAATGAACCAGATAC | |
| Gabpa-F | GAACGTCTTCAACCATGAC | |
| Gabpa-R | CTAGGTCTCAAATCTCTTTGTC | |
| **ChIP assay** | | |
| Sqle | CCTGTAGCTCTCTTGCGTTTGA | GCTCGCTCTGGAGGAACTCTT |
